# Short dual antiplatelet therapy and dual antiplatelet therapy de-escalation after primary percutaneous intervention: For whom and how

**DOI:** 10.3389/fcvm.2022.1008194

**Published:** 2022-11-10

**Authors:** Marie Muthspiel, Christoph C. Kaufmann, Achim Leo Burger, Benjamin Panzer, Freek W. A. Verheugt, Kurt Huber

**Affiliations:** ^1^Cardiology and Intensive Care Medicine, Clinic Ottakring (Wilhelminenhospital), Vienna, Austria; ^2^Department of Cardiology, Onze Lieve Vrouwe Gasthuis, Amsterdam, Netherlands; ^3^Medical Faculty, Sigmund Freud University, Vienna, Austria

**Keywords:** percutaneous coronary intervention (PCI), short dual antiplatelet therapy (short DAPT), de-escalation, P2Y_12_-inhibitor, bleeding

## Abstract

Dual antiplatelet therapy (DAPT) for 6–12 months, followed by lifelong aspirin monotherapy is considered an effective standard therapy for the prevention of thrombo-ischemic events in patients with acute and chronic coronary syndrome (ACS, CCS) undergoing percutaneous coronary intervention (PCI) or after a primarily conservative treatment decision. In ACS patients, the stronger P2Y_12_-inhibitors ticagrelor or prasugrel are recommended in combination with aspirin unless the individual bleeding risk is high and shortening of DAPT is warranted or clopidogrel is preferred. However, also in patients at low individual bleeding risk, DAPT is associated with a higher risk of bleeding. In recent years, new antithrombotic treatment strategies, such as shortening DAPT followed by early P2Y_12_-inhibitor monotherapy and de-escalating DAPT from potent P2Y_12_-inhibitors to clopidogrel by maintaining DAPT duration time, have been investigated in clinical trials and shown to reduce bleeding complications in cardiovascular high-risk patients without negative effects on ischemic events. In this review, we summarize the current knowledge and discuss its implication on future antithrombotic strategies in terms of a personalized medicine.

## Introduction

**FIGURE 1:**
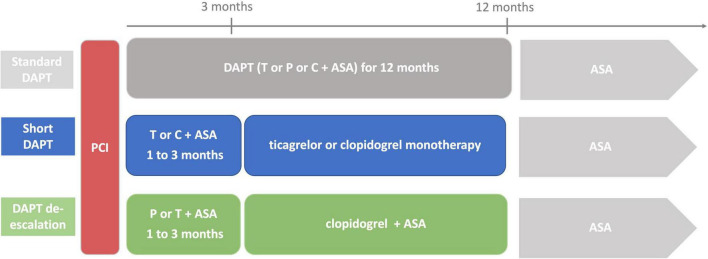
Antithrombotic strategies of Standard DAPT, Short DAPT and DAPT De-escalation. DAPT: dual antiplatelet therapy, T: Ticagrelor, P: Prasugrel, C: Clopidogrel, ASA: Acetylsalicylic Acid.

Dual antiplatelet therapy (DAPT) is the cornerstone in the prevention of thrombo-ischemic events in patients with acute and chronic coronary syndrome (ACS, CCS) after primary percutaneous intervention (PCI). European Society of Cardiology (ESC) guidelines recommend a combination of aspirin with ticagrelor or with prasugrel for a period of 6 (CCS) to 12 (ACS) months, followed by a lifelong aspirin monotherapy in patients with low bleeding risk (Class Ia indication) (1, 2). DAPT duration should be adjusted to the individual patient’s bleeding risk using appropriate risk scores, such as the DAPT and the PRECISE-DAPT score. In patients at high bleeding risk (HBR), DAPT can be shortened (< 6 or < 12 months) by early withdrawal of the P2Y_12_-inhibitor (1, 2). In patients with high ischemic risk and without increased risk of major bleeding, DAPT can be extended (> 12 months) after ACS (1). However, standard DAPT has been shown to effectively reduce major adverse cardiovascular events (MACE) in patients after PCI but is associated with increased risk of bleeding (3). Accordingly, safe antiplatelet strategies reducing bleeding rates but without adverse effects on ischemic outcomes are mandatory. To address this issue, new antithrombotic treatment strategies for cardiovascular high-risk patients have been evolved and investigated in clinical trials in recent years. In this review, we focus on the current knowledge of short DAPT followed by early P2Y_12_-inhibitor monotherapy and on DAPT de-escalation from potent P2Y_12_-inhibitors to clopidogrel in terms of a personalized medicine (Figure 1).

## P2Y12-inhibitor monotherapy after short dual antiplatelet therapy

According to ESC guidelines, short DAPT originally consists of early P2Y_12_-inhibitor withdrawal and subsequent lifelong aspirin monotherapy, as it should be considered for non-ST-elevation (NSTE)-ACS patients with stent implantation who are at high risk of bleeding (1). More recently, however, short DAPT refers to discontinuation of aspirin in favor of monotherapy with a strong oral P2Y_12_-inhibitor after an initial 1–3-month period of DAPT. This intriguing treatment approach presents a promising option to reduce bleeding risk in CCS and ACS patients after PCI and has recently been investigated in several trials (4–10).

Moreover, three meta-analyses demonstrated that withdrawal of aspirin in favor of P2Y_12_-inhibitor monotherapy after 1–3 months of DAPT significantly reduced the risk of major bleeding without increasing ischemic endpoints (11–13). In ACS patients, P2Y_12_-inhibitor monotherapy reduced bleeding risk by 50% (HR 0.50, 95% CI 0.41–0.61, *p* < 0.001) with no significant change in MACE rates when compared with standard DAPT (HR 0.85, 95% CI 0.70–1.0, *p* = 0.09) (12). Current data highlight the large contribution of aspirin to the bleeding risk of DAPT (13). However, it is important to note that between trials, patient populations differed in terms of their bleeding risk and selection of P2Y_12_-inhibitor.

### Trials on P2Y_12_-inhibitor monotherapy after short dual antiplatelet therapy

#### With clopidogrel

Three large scaled randomized controlled trials (RCTs) provide 1-year data on clopidogrel monotherapy after short DAPT in patients undergoing PCI (Table 1) (4–6). In a population of CCS (58%) and ACS (42%) patients at low-to-moderate bleeding risk, the results of the Effect of P2Y_12_ Inhibitor Monotherapy vs. Dual Antiplatelet Therapy on Cardiovascular Events in Patients Undergoing Percutaneous Coronary Intervention (SMART-CHOICE) trial showed, that clopidogrel monotherapy after 3 months of DAPT was non-inferior to standard DAPT with respect to the primary ischemic endpoint (all-cause death, MI, or stroke) (95% CI –∞-1.3%, *p*_non–inferiority_ = 0.007) (4). In addition, the short DAPT strategy resulted in significantly lower bleeding rates [Bleeding Academic Research Consortium (BARC) 2–5] when compared with standard therapy (2.0% vs. 3.4%, HR 0.58, 95% CI 0.36–0.92, *p* = 0.002) (4).

**TABLE 1:** Baseline characteristics of randomized-controlled trials on short dual antiplatelet therapy following clopidogrel monotherapy.

Study	*n*	Ischemic and bleeding risk	Ethnicity	Clinical setting (%)		DAPT duration	Follow-up (months)	Primary endpoint	Primary endpoint met
				ACS	CCS				
SMART-CHOICE	2,993	Low-to-moderate bleeding risk, low-to-moderate ischemic risk	East Asian	58	42	3 vs. 12 months	12	All-cause death, MI, or stroke	Yes
STOPDAPT-2	3,045	Low-to-moderate bleeding risk, low-to-moderate ischemic risk	East Asian	38	62	1 vs. 12 months	12	CV death, MI, ST, stroke, or TIMI major or minor bleeding	Yes
STOPDAPT-2-ACS	4,169	Low-to-moderate bleeding risk, mixed ischemic risk*	East Asian	100	0	1 vs. 12 months	12	CV death, MI, ST, stroke, or TIMI major or minor bleeding	NO
MASTER-DAPT	4,434	HBR, mixed ischemic risk*	Caucasian	49	51	1 vs. ≥3 months	11	All-cause death, MI, stroke, or BARC type 3, or 5	Yes

n, number of patients; ACS, acute coronary syndrome; CCS, chronic coronary syndrome; DAPT, dual antiplatelet therapy; HBR, high bleeding risk; MI, myocardial infarction; CV, cardiovascular; ST, stent thrombosis; TIMI, Thrombolysis in Myocardial Infarction; BARC, Bleeding Academic Research Consortium. *Mixed ischemic risk includes low, moderate and high ischemic risk.

Moreover, the Japanese Results of the Effect of 1-month Dual Antiplatelet Therapy followed by Clopidogrel vs. 12-month Dual Antiplatelet Therapy on Cardiovascular and Bleeding Events in Patients receiving PCI (STOPDAPT-2) trial indicated that in ACS (38%) and CCS (62%) patients at low-to-moderate bleeding risk, clopidogrel monotherapy following an even shorter DAPT period of 1 month reduced bleeding rates [Thrombolysis in Myocardial Infarction (TIMI) major or minor bleedings] without causing a significant increase in primary combined endpoint event rates [cardiovascular (CV) death, myocardial infarction (MI), stent thrombosis (ST), stroke or TIMI major or minor bleeding] (HR 0.26, 95% CI 0.11–0.64, *p* = 0.004 and HR 0.64, 95% CI 0.42–0.98, *p* = 0.04, respectively) (5). While these data have shown safety for very early clopidogrel monotherapy in predominantly stable patients, the results of the STOPDAPT-2-ACS trial suggest, that this does not apply to unstable patients (6). This study, enrolling only ACS patients (*n* = 4,169), failed to meet their primary non-inferiority endpoint (of CV death, MI, ST, stroke or TIMI major or minor bleeding) at 12 months (HR 1.14, 95% CI 0.80–1.6, *p*_non–inferiority_ = 0.06) (6). Patients in the short DAPT group further presented a numerically but not significantly higher incidence of the major secondary cardiovascular endpoints than patients treated with standard DAPT (2.76% vs. 1.86%, HR 1.50 95% CI 0.99–2.26) (6).

The aforementioned trials show that clopidogrel monotherapy after short DAPT presents a safe therapeutic option to reduce bleeding rates in stable patients at low-to-moderate bleeding risk and mixed ischemic risk (includes low, moderate, and high ischemic risk). However, these data do not extend to patients in high-risk settings. In this regard, the High Bleeding Risk Patients Post Bioresorbable Polymer Coated Stent Implantation with an Abbreviated vs. Standard DAPT Regimen (MASTER-DAPT) trial was the first to selectively include patients at high bleeding risk, demonstrating that even in these patients, short DAPT followed by single antiplatelet therapy is a safe strategy to prevent bleeding after PCI (Table 1) (7). Specifically, 1-month DAPT proved non-inferior to standard DAPT in terms of the primary combined endpoint (all-cause death, MI, stroke, BARC type 3, or 5) and was associated (95% CI:-1.80 to 33, *p*_non–inferiority_ < 0.001) with a lower incidence of major or clinically relevant non-major bleedings (BARC type 2, 3, or 5) (6.5% vs. 9.11%, 95% CI:-4.40 to 1.24, *p*_non–inferiority_ < 0.001) at 11 months (7).

#### With ticagrelor

The GLOBAL LEADERS (Ticagrelor plus aspirin for 1 month, followed by ticagrelor monotherapy for 23 months vs. aspirin plus clopidogrel or ticagrelor for 12 months, followed by aspirin monotherapy for 12 months after implantation of a drug-eluting stent) trial compared 1-month DAPT followed by ticagrelor monotherapy with 12 months of DAPT after PCI. In this trial, patients at low bleeding risk and mixed ischemic risk were included (Table 2) (8). The trial failed to show that 23-month ticagrelor monotherapy after short DAPT was associated with lower primary endpoint events (all-cause death, MI) (RR 0.87, 95% CI 0.75–1.01, *p* = 0.073). However, non-inferiority was met and bleeding rates (BARC type 3, or 5) were similar between groups (2.04% vs. 2.12%, RR 0.97, 95% CI 0.78–1.20, *p* = 0.77) (8). Consistent findings with respect to the primary efficacy and safety endpoints were demonstrated in the prespecified GLOBAL LEADERS Adjudication Sub-Study (GLASSY) (9). Moreover, a 31% relative risk reduction in urgent target vessel revascularization (TVR) (1.87% vs. 2.72%, RR 0.69, 95% CI 0.51–0.93) was found in the experimental arm and shown to increase consistently over time (9). Short DAPT was further associated with lower rates of MI (RR 0.54, 95% CI 0.33–0.88, *p*_interaction_ = 0.062) and ST (RR 0.14, 95% CI 0.03–0.63; *p*_interaction_ = 0.007) at 12-month follow-up, indicating that ticagrelor monotherapy may have beneficial effects on the occurrence of MI and ST when compared with aspirin alone (9).

**TABLE 2:** Baseline characteristics of randomized-controlled trials on short DAPT following ticagrelor monotherapy.

Study	*n*	Bleeding and ischemic risk	Ethnicity	Clinical setting (%)		DAPT duration	Follow-up (months)	Primary endpoint	Primary endpoint met
				ACS	CCS				
GLOBAL-LEADERS	15,968	Low bleeding risk, mixed ischemic risk*	Caucasian	47	53	1 vs. 12 months	24	All-cause death, MI	NO
TWILIGHT	7,119	Low bleeding risk, mixed ischemic risk	Caucasian East Asian	64	36	3 vs. 12 months	15	BARC type 2,3, or 5	Yes
TICO	3,056	Low bleeding risk, mixed ischemic risk	East Asian	100	0	3 vs. 12 months	12	TIMI major bleeding, all-cause death, MI, ST, stroke, or TVR	Yes

n, number of patients; ACS, acute coronary syndrome; CCS, chronic coronary syndrome; DAPT, dual antiplatelet therapy; MI, Myocardial infarction; BARC, Bleeding Academic Research Consortium; TIMI, Thrombolysis in Myocardial Infarction; ST, stent thrombosis; TVR, target vessel revascularization. *Mixed ischemic risk includes low, moderate and high ischemic risk.

In The Ticagrelor with or without Aspirin in High-Risk Patients after PCI (TWILIGHT) trial, 7,119 patients (64% ACS, 36% CCS) at low bleeding and mixed ischemic risk were enrolled (10). Ticagrelor monotherapy after DAPT of 3 months was associated with a 44% lower risk of bleeding (BARC type 2,3, or 5) than standard DAPT (HR 0.56, 95% CI 0.45–0.68, *p* < 0.001) with no significant increase in MACE (death, MI, stroke) (HR 0.99, 95% CI 0.78–1.25, *p*_non–inferiority_ < 0.001) over 15 months after PCI (10). Several prespecified subgroup-analyses (patients at HBR, ACS, complex PCI, diabetes, gender) demonstrated comparable outcomes (11–15).

Further, the Ticagrelor Monotherapy vs. Dual-Antiplatelet Therapy After PCI (SIDNEY) meta-analysis, including data from GLASSY and TWILIGHT, provides strong evidence for the reduction of bleeding rates with ticagrelor monotherapy (16).

Focusing on unstable patients after PCI, Franzone et al. demonstrated that safety effects of ticagrelor monotherapy after 1-month DAPT on ischemic endpoints were consistent in patients with or without ACS, but only ACS patients had a net clinical benefit in regards of a composite endpoint of both co-primary study endpoints from GLASSY (17). The South Korean Effect of Ticagrelor Monotherapy vs. Ticagrelor With Aspirin on Major Bleeding and Cardiovascular Events in Patients With Acute Coronary Syndrome (TICO) trial was the only trial to prospectively investigate ticagrelor monotherapy exclusively in ACS patients (18, 19). Switching to ticagrelor monotherapy after 3 months of DAPT significantly reduced primary adverse clinical events (TIMI major bleeding, all-cause death, MI, ST, stroke, TVR) (HR 0.66, 95% CI 0.48–0.92, *p* = 0.01) and was associated with lower risk of major bleeding (HR 0.56, 95% CI 0.34–0.91, *p* = 0.02) (18). Importantly, only patients at low bleeding risk were included in this trial. The results from the STOPDAPT-2-ACS and TICO trials suggest that ticagrelor but not clopidogrel monotherapy presents a safe antiplatelet treatment regimen for ACS patients after short DAPT. Therefore, it has been discussed whether clopidogrel monotherapy initiated 1 month after DAPT is less effective in ACS patients due to the increased ischemic risk up to 3 months post ACS and the lower P2Y_12_-inhibiting capacity of the agent.

#### With prasugrel

The clinical benefit of prasugrel monotherapy in patients after PCI has not been sufficiently investigated to date. The Aspirin-free Prasugrel Monotherapy Following Coronary Artery Stenting in Patients with Stable CAD (ASET) study assessed prasugrel monotherapy (60 mg loading dose followed by 10 mg/day) after PCI in 202 CCS patients at low ischemic risk. Until PCI, patients received clopidogrel-based DAPT. At 3 months of follow-up, there was no primary endpoint event and only one fatal intracranial hemorrhage 6 h after PCI (20).

### Ongoing trials on P2Y_12_-inhibitor monotherapy and short dual antiplatelet therapy

Several ongoing trials are addressing the above-mentioned issues through different approaches.

The Ticagrelor Monotherapy in Patients Treated With New-generation Drug-eluting Stents for Acute Coronary Syndrome (T-PASS) (NCT03797651) trial evaluates ticagrelor monotherapy following very-short DAPT less than 1 month after PCI in ACS patients.

Results from the A Randomized Comparison of Clopidogrel Monotherapy vs. Extended Dual-antiplatelet Therapy Beyond 12 Months After Implantation of Drug-eluting Stents in High-risk Lesions or Patients trial (A-CLOSE) (NCT03947229) are expected at the end of 2023, investigating clopidogrel monotherapy vs. extended clopidogrel-based DAPT from 12 to 36 months after PCI in patients at high risk for either ischemic or bleeding complications.

The P2Y_12_-Inhibitor Monotherapy vs. Extended DAPT in Patients Treated With Bioresorbable Scaffold trial (SMART-CHOICE II) (NCT03119012) currently compares clopidogrel or ticagrelor monotherapy from 12 to 36 months after PCI with extended ticagrelor-based DAPT for 36 months after PCI. Long-term clopidogrel monotherapy vs. aspirin monotherapy after 12 months of DAPT is currently being investigated in patients at high risk for recurrent ischemic events in The Choice of Optimal Anti-Thrombotic Strategy in Patients Undergoing Implantation of Coronary Drug-Eluting Stents 3 trial (SMART-CHOICE III) (NCT04418479).

Further data on prasugrel monotherapy in CCS and non-STE elevation ACS (NSTE-ACS) patients are expected from the still ongoing Acetyl Salicylic Elimination Trial JAPAN (ASET-JAPAN pilot study) (NCT 05117866) in 2024.

## Dual antiplatelet therapy de-escalation strategies

The benefit of potent P2Y_12_-inhibitors regarding ischemic risk reduction is greatest during the acute and sub-acute phase after the index event whilst bleeding risk persists during maintenance therapy (21, 22). Hence, in a significant amount (up to 28%) of ACS patients, physicians tend to switch from standard DAPT by using ticagrelor or prasugrel in association with aspirin to clopidogrel and aspirin within 1 year after PCI (23). Besides economic factors, bleeding complications are the most common reason for a so-called DAPT de-escalation (24). Clinical data justifying this strategy have long been limited. However, in recent years, different studies have provided data on the safety and efficacy of switching to clopidogrel after a short period of DAPT with potent P2Y_12_-inhibitors in ACS patients (Table 3) (25–28).

**TABLE 3:** Baseline characteristics of randomized-controlled trials on DAPT de-escalation.

Study	*n*	Bleeding and ischemic risk	Ethnicity	Clinical setting (%)		De-escalation strategy	Timing of de-escalation	Follow-up (months)	Primary endpoint	Primary endpoint met
				ACS	CCS					
**Unguided de-escalation**										
TOPIC	646	Low-to-moderate bleeding risk, mixed ischemic risk*	Caucasian	100	0	Clopidogrel-based DAPT vs. ticagrelor-/prasugrel-based DAPT	30 days after PCI	12	CV death, TVR, stroke, BARC type ≥ 2	Yes
TALOS-AMI	2,697	Low-to-moderate bleeding risk, mixed ischemic risk	East Asian	100	0	Clopidogrel-based DAPT vs. ticagrelor-based DAPT	30 days after PCI	12	CV death, MI, stroke, BARC type 2,3, or 5	Yes
**Guided de-escalation**										
TROPICAL-ACS	2,610	Low-to-moderate bleeding risk, mixed ischemic risk	Caucasian	100	0	Clopidogrel-based DAPT vs. prasugrel-based DAPT	Days 7–14 after discharge	12	CV death, MI, stroke, BARC type ≥ 2	Yes
POPULAR GENETICS	2,488	Low-to-moderate bleeding risk, mixed ischemic risk	Caucasian	100	0	Clopidogrel-based DAPT vs. ticagrelor-based DAPT	Day 1–3 after PCI	12	All-cause death, MI, ST, stroke, or PLATO major bleeding;	Yes

n, number of patients; ACS, acute coronary syndrome; CCS, chronic coronary syndrome; STEMI, ST-elevation myocardial infarction; NSTEMI, non-ST-elevation myocardial infarction; DAPT, dual antiplatelet therapy; PCI, percutaneous coronary intervention; CV, cardiovascular; TVR, target vessel revascularization; BARC, Bleeding Academic Research Consortium; MI, Myocardial infarction; PFT, Platelet Function Testing; ST, stent thrombosis; PLATO, Platelet Inhibition and Patient Outcomes. *Mixed ischemic risk includes low, moderate and high ischemic risk.

The monocentric, randomized, open-label Timing of Platelet Inhibition After Acute Coronary Syndrome (TOPIC) trial investigated DAPT de-escalation in 646 ST-elevation myocardial infarction (STEMI) and non-ST-elevation myocardial infarction (NSTEMI) patients at low-to-moderate bleeding risk (26). At 1 month after PCI, patients in the experimental group were switched to clopidogrel-based DAPT while standard DAPT was maintained in the control group. At 12 months after the index event, the de-escalation strategy was superior to standard DAPT in terms of the primary combined endpoint (CV death, urgent revascularization, stroke, BARC type ≥ 2) (HR 0.48 95% CI 0.34–0.68, *p* < 0.01) and was further associated with a lower risk of bleeding (BARC type ≥ 2) (HR 0.30, 95% CI 0.18–0.50, *p* < 0.01) (26).

Similarly, results of the multicentric, randomized Ticagrelor vs. Clopidogrel in Stabilized Patients with Acute Myocardial Infarction (TALOS-AMI) study have shown that unguided DAPT de-escalation was associated with a 45% lower risk of net clinical events (CV death, MI, stroke, BARC type 2,3,5) (HR 0.55, 95% CI 0.40–0.76, *p*_non–inferiority_ < 0.001) and with reduced risk of bleeding complications (BARC type 2,3, or 5) (HR 0.52, 95% CI 0.35–0.77, *p* = 0.0012) when compared with ticagrelor-based DAPT at 12 months after PCI (27). However, only South Korean STEMI and NSTEMI patients at low-to-moderate bleeding risk were included. The validity of these data must therefore be qualified for Caucasian populations due to the higher prevalence of *CYP2C19* loss-of-function alleles in the East Asian population (29).

Recently, two large-scaled RCTs provided data on clopidogrel-based DAPT in Caucasian ACS patients (25, 28). Both trials performed platelet function- and genetic-directed de-escalation, respectively. In the Testing Responsiveness to Platelet Inhibition On Chronic Antiplatelet Treatment For Acute Coronary Syndromes (TROPICAL-ACS) trial, 2,610 STEMI and NSTEMI patients after PCI were treated with standard therapy consisting of prasugrel and aspirin (25). Seven days after discharge, patients randomized to the de-escalation group were switched to clopidogrel for another 7 days whereas standard DAPT was maintained in the control group. Platelet Function Testing (PFT) was performed on day 14 to identify clopidogrel non- or low-responder patients and readjust them to prasugrel. At 12 months follow-up, clopidogrel-based DAPT was not inferior to standard DAPT in terms of the primary combined endpoint (CV death, MI, stroke, BARC type ≥ 2) (HR 0.81, 95% CI 0.62–1.06, *p*_non–inferiority_ = 0.0004) (25). Despite a trend toward lower bleeding risk in the de-escalation group, bleeding rates (BARC type ≥ 2) did not significantly differ between groups (5% vs. 6%, HR 0.82, 95% CI 0.59–1.13, *p* = 0.23) (25).

In the Genotype-Guided Strategy for Oral P2Y_12_ Inhibitors in Primary PCI (POPULAR GENETICS) trial *CYP2C19*-directed genetic testing was used as safety tool in 2488 STEMI patients (28). Within 3 days after PCI, non-carriers received clopidogrel-based DAPT whereas carriers of CYP2C19*2 or CYP2C19*3 LOF-alleles received standard DAPT with prasugrel or ticagrelor (28). The results demonstrated non-inferiority of guided de-escalation in terms of net clinical events (all-cause death, MI, ST, stroke, Platelet Inhibition and Patient Outcomes (PLATO) major bleeding) (95% CI 2.0–0.7, *p*_non–inferiority_ < 0.001) as well as significantly lower bleeding rates (PLATO major or minor bleeding) compared to standard DAPT at 12-month follow-up (HR 0.78, 95% CI 0.61–0.98, *p* = 0.04) (28). However, it should be noted that patients enrolled in the TROPICAL-ACS and POPULAR GENETICS trials only had low-to-moderate bleeding and mixed ischemic risk.

Finally, two recent meta-analyses demonstrated overall efficacy and safety of DAPT de-escalation (30, 31). In one study, guided selection of antiplatelet therapy after PCI was shown to reduce the risk of MACE (RR 0.78, 95% CI 0.63–0.95, *p* = 0.015) without any trade-off in bleeding rates when compared with standard DAPT (RR 0.88, 95% CI 0.77–1.01, *p* = 0.069) (30). Importantly, this analysis included studies that investigated both de-escalation and escalation strategies in ACS and CCS patients. The other meta-analysis by Tavenier et al. exclusively focused on RCTs that studied DAPT de-escalation in ACS patients (31). The results demonstrated that a strategy of de-escalation vs. standard DAPT reduces both clinically relevant bleedings (BARC type ≥ 2) (HR 0.57, 95% CI 0.42–0.78) and MACE rates (HR 0.77, 95% CI 0.62–0.96) (31).

### Antiplatelet responsiveness of clopidogrel

When using clopidogrel, interpatient variability in antithrombotic efficacy must be considered (32, 33). Genetic and metabolic factors influence pharmacologic response to clopidogrel resulting in increased ischemic risk in certain patients (32). In the TROPICAL-ACS and POPULAR GENETICS trials, PFT and genetic testing were used as safety tools, whereas unguided de-escalation was performed in the TOPIC and TALOS-AMI trials. According to ESC guidelines, routine use of PFT or genetic testing in the selection of antiplatelet therapy is not recommended. When de-escalation to clopidogrel is performed, the strategy (guided vs. unguided) should be determined based on the patient’s risk profile and the availability of respective assays (1). However, guidelines do not specify in which patients a guided approach should be considered, leaving this decision to the treating physician. In this regard, an expert consensus from 2019 provides more detailed recommendations (34). Unfortunately results from the POPULAR GENETICS trial and from more recent meta-analyses were not included at this time but are considered in the present work.

Tavenier et al. demonstrated that significant bleeding risk reduction was consistent in both guided (HR 0.79, 95% CI 0.66–0.94) and unguided (HR 0.44, 95% CI 0.32–0.59) de-escalation (31). Interestingly, unguided de-escalation was associated with a greater reduction of bleeding risk (*p*_interaction_ = 0.037) when compared with guided de-escalation (31). However, the authors noted that the bleeding benefit may be explained by the fact that the proportion of patients who received clopidogrel was higher in the unguided than in the guided de-escalation group (31).

If PFT or genetic testing is considered, limitations of the respective test methods must be taken into account (Table 4). PFT results vary significantly depending on the different assays available (VerifyNow, Multiplate, VASP, TEG platelet mapping), which not only makes it difficult to compare data from different studies but may also influence clinical decisions (35). Conversely, there are no relevant discrepancies between validated genetic assays (32). Since PFT can only be performed on-treatment, initial clopidogrel therapy and, if needed, subsequent medication adjustment is required (28). This could affect patient compliance.

**TABLE 4:** Advantages and disadvantages of platelet function vs. CYP2C19-directed genetic testing, modified after Sibbing et al. (34).

	PFT	Genetic testing
Availability of different assays	Yes	Yes
Absence of interassay variability	No	Yes
No need to perform on-treatment	No	Yes
Assessment of non-genetic factors	Yes	No
Direct measurement of treatment response	Yes	No
Absence of temporal variability	No	Yes

PFT, platelet function testing.

In the TROPICAL-ACS trial, patients received clopidogrel treatment from days 7 to 14 after hospital discharge (25). Given the increased ischemic risk in the acute and subacute phase after the index event, early clopidogrel therapy may not guarantee adequate platelet inhibition in patients on high on-treatment platelet reactivity (HPR) (24, 36).

*CYP2C19* genetic testing, as performed in the POPULAR GENETICS trial, does not require clopidogrel treatment. However, it does not directly reflect treatment response as various intrinsic and extrinsic factors that influence clopidogrel efficacy (BMI ≥ 30 kg/m^2^, diabetes mellitus, gastrointestinal absorption, drug interactions, patient adherence) are not taken into account (37, 38). It should also be noted that a single platelet function test only reflects the current status of response to treatment, and the optimal timing of measurement is unknown (34).

In this regard, a pre-specified TROPICAL-ACS sub-study showed that platelet reactivity during clopidogrel therapy is subject to diurnal variability, with a peak in platelet reactivity at the end of the dosing interval (39). However, clinical outcomes of these findings have not been investigated. Since these epigenetic factors vary over time, it could be questioned whether a single measurement is sufficient or whether PFT should be repeated during maintenance therapy.

To summarize, one meta-analysis provides data suggesting a greater reduction in bleeding risk with unguided vs. guided de-escalation and similar ischemic risk reduction with both strategies (31). Current knowledge does not show superiority of specific assays (PFT vs. *CYP2C19* genetic testing) and therefore respective limitations should be considered when de-escalation to clopidogrel is performed.

## Implications of short dual antiplatelet therapy and P2Y_12_-inhibitor de-escalation strategies for future antiplatelet therapy

Both withdrawal of aspirin 1–3 months after PCI with continued use of P2Y_12_-inhibitor monotherapy and de-escalation of P2Y_12_-inhibitor therapy by switching from more potent inhibitors to clopidogrel reduce bleeding risk without any trade-off in MACE when compared with standard DAPT. These findings now raise the question of which patient populations may benefit from a personalized antiplatelet strategy and how early aspirin should be discontinued or ticagrelor or prasugrel replaced with clopidogrel.

### Dual antiplatelet therapy de-escalation

Based on the positive results of the TROPICAL-ACS trial, ESC Guidelines recommend guided or unguided de-escalation as an alternative treatment regimen in ACS patients who are not suitable for 12 months potent platelet inhibition (Class IIb indication) (25). However, guidelines do not comment on the timing of DAPT de-escalation. Trials investigating guided de-escalation (TROPICAL-ACS, POPULAR GENETICS) switched to clopidogrel maintenance therapy within 14 days after PCI (25, 28). Unguided de-escalation was performed at 1 month after PCI in the TOPIC and TALOS-AMI trials (26, 27). Given the highest ischemic risk in the first month after the index event, the timing of unguided de-escalation seems to be appropriate to prevent recurrence of ischemic events (24, 36). A guided approach, in turn, allows the identification of non- or low-responder patients at high ischemic risk at an early stage and seems to justify the timing of de-escalation. The above-mentioned trials have shown short-term safety of DAPT de-escalation for ACS patients at low-to-moderate bleeding risk but clinical outcomes beyond 1 year have not been investigated yet. Further, the studies were not adequately powered with respect to primary ischemic endpoints. Finally, it must be noted that patients at high thrombotic risk (HTR) may not have been adequately enrolled and the results may apply only to populations with balanced ischemic risk. To the best of our knowledge, no ongoing trials are currently addressing these issues.

### P2Y_12_-inhibitor monotherapy after short dual antiplatelet therapy

Results on short DAPT had already an impact on recent guidelines recommending early P2Y_12_-inhibitor monotherapy in certain patient populations (40).

The MASTER-DAPT trial has shown that also patients at high bleeding risk are suitable for clopidogrel monotherapy after short DAPT. However, certain patient populations, like elderly patients, were underrepresented in the above-mentioned trials. This population, which bears an increased bleeding risk in itself, might be suitable for short DAPT or de-escalation as recently discussed in an editorial of the European Heart Journal (41).

While trials have provided evidence on patients with low-to-moderate and high bleeding risk, no clear conclusion can be drawn from existing data for the treatment of patients at high thrombotic risk. This could be due to inclusion bias in the existing studies, as HTR patients are not represented in sufficient numbers.

Ticagrelor but not clopidogrel monotherapy after very short DAPT has been shown to be a safe strategy to treat ACS patients. Therefore, it has been discussed whether clopidogrel monotherapy initiated 1 month after DAPT is less effective due to the increased ischemic risk in the early phase after ACS and the lower P2Y_12_-inhibitory capacity of the agent. Here, DAPT of up to 3 months, measurement of response to clopidogrel, or switching to a more potent P2Y_12_-inhibitor as monotherapy (theoretically, as not yet tested) might be useful to keep the rate of ischemic events low. Nevertheless, existing data only apply for ACS patients at low-to-moderate bleeding risk since ACS patients at HBR were not included in previous trials.

It is currently unclear whether P2Y_12–_inhibitor monotherapy should be prolonged (which was done for 24 months in the GLOBAL LEADERS trial), switched back to aspirin lifelong after 12 months (as done in TWILIGHT and TICO), or switched to clopidogrel lifelong, as shown in a recent study (42). In the Aspirin vs. Clopidogrel for Chronic Maintenance Monotherapy after Percutaneous Coronary Intervention (HOST-EXAM) trial, more than 5,000 patients after PCI, who received 6–18 months of DAPT, were investigated (42). Subsequent clopidogrel monotherapy resulted in a significant reduction in the net clinical endpoint (death, MI, insult, ACS, BARC type 3, or 5) when compared with aspirin monotherapy at 24 months (HR = 0.73, 95% CI: 0.59–0.90, *p* = 0.0035) (42).

## Summary

The use of short DAPT and DAPT de-escalation is currently limited to HBR patients, while patients at normal-to-low bleeding risk usually continue to be treated with standard DAPT. To determine a tailored antiplatelet regimen that strikes the balance between bleeding risk reduction and prevention of recurrent ischemic events, several trials have shown efficacy and safety of DAPT de-escalation and P2Y_12_-inhibitor monotherapy after short DAPT. However, based on inclusion bias, patients at very high thrombotic risk only represent a low percentage in the aforementioned trials and elderly patients have not been included in sufficient numbers. Especially, for elderly with no additional risk factors other than their age, the new antiplatelet strategies of short DAPT and DAPT de-escalation might be of interest for future clinical practice. Prospective RCTs in specific patient groups and long-term safety data regarding hard ischemic endpoints are pending which still limits broad use of short DAPT and DAPT de-escalation in patients after PCI. Finally, personalized antiplatelet treatment should equally consider the patient’s ischemic and bleeding risk and in case clopidogrel is used, potential interindividual differences in platelet responsiveness must always be taken into account.

## Author contributions

MM and KH worte the manuscript. All authors contributed to the article and approved the submitted version.

## References

[1] Collet JP Thiele H Barbato E Barthélémy O Bauersachs J Bhatt DL 2020 ESC guidelines for the management of acute coronary syndromes in patients presenting without persistent ST-segment elevation. *Eur Heart J.* (2021) 42:1289–367. 10.1093/eurheartj/ehaa575 32860058

[2] Neumann FJ Sousa-Uva M Ahlsson A Alfonso F Banning AP Benedetto U 2018 ESC/EACTS guidelines on myocardial revascularization. *Eur Heart J.* (2019) 40:87–165. 10.1093/eurheartj/ehy394 30165437

[3] Navarese EP Andreotti F Schulze V Kołodziejczak M Buffon A Brouwer M Optimal duration of dual antiplatelet therapy after percutaneous coronary intervention with drug eluting stents: meta-analysis of randomised controlled trials. *BMJ.* (2015) 350:h1618. 10.1136/bmj.h1618 25883067 PMC4410620

[4] Hahn JY Song YB Oh JH Chun WJ Park YH Jang WJ Effect of P2y12 inhibitor monotherapy vs dual antiplatelet therapy on cardiovascular events in patients undergoing percutaneous coronary intervention: the smart-choice randomized clinical trial. *JAMA.* (2019) 321:2428–37. 10.1001/jama.2019.8146 31237645 PMC6593635

[5] Watanabe H Domei T Morimoto T Natsuaki M Shiomi H Toyota T Effect of 1-month dual antiplatelet therapy followed by clopidogrel vs 12-month dual antiplatelet therapy on cardiovascular and bleeding events in patients receiving PCI: the stopdapt-2 randomized clinical trial. *JAMA.* (2019) 321:2414–27. 10.1001/jama.2019.8145 31237644 PMC6593641

[6] Watanabe H Morimoto T Natsuaki M Yamamoto K Obayashi Y Ogita M Comparison of clopidogrel monotherapy after 1 to 2 months of dual antiplatelet therapy with 12 months of dual antiplatelet therapy in patients with acute coronary syndrome: the stopdapt-2 ACS randomized clinical trial. *JAMA Cardiol.* (2022) 7:407–17. 10.1001/jamacardio.2021.5244 35234821 PMC8892373

[7] Valgimigli M Frigoli E Heg D Tijssen J Jüni P Vranckx P Dual antiplatelet therapy after PCI in patients at high bleeding risk. *N Engl J Med.* (2021) 385:1643–55. 10.1056/NEJMoa2108749 34449185

[8] Vranckx P Valgimigli M Jüni P Hamm C Steg PG Heg D Ticagrelor plus aspirin for 1 month, followed by ticagrelor monotherapy for 23 months vs aspirin plus clopidogrel or ticagrelor for 12 months, followed by aspirin monotherapy for 12 months after implantation of a drug-eluting stent: a multicentre, open-label, randomised superiority trial. *Lancet.* (2018) 392:940–9. 10.1016/s0140-673631858-0 30166073

[9] Franzone A McFadden E Leonardi S Piccolo R Vranckx P Serruys PW Ticagrelor alone versus dual antiplatelet therapy from 1 month after drug-eluting coronary stenting. *J Am Coll Cardiol.* (2019) 74:2223–34. 10.1016/j.jacc.2019.08.1038 31672177

[10] Mehran R Baber U Sharma SK Cohen DJ Angiolillo DJ Briguori C Ticagrelor with or without aspirin in high-risk patients after PCI. *N Engl J Med.* (2019) 381:2032–42. 10.1056/NEJMoa1908419 31556978

[11] Angiolillo DJ Baber U Sartori S Briguori C Dangas G Cohen DJ Ticagrelor with or without aspirin in high-risk patients with diabetes mellitus undergoing percutaneous coronary intervention. *J Am Coll Cardiol.* (2020) 75:2403–13. 10.1016/j.jacc.2020.03.008 32240760

[12] Vogel B Baber U Cohen DJ Sartori S Sharma SK Angiolillo DJ Sex differences among patients with high risk receiving ticagrelor with or without aspirin after percutaneous coronary intervention: a subgroup analysis of the twilight randomized clinical trial. *JAMA Cardiol.* (2021) 6:1032–41. 10.1001/jamacardio.2021.1720 33991416 PMC8124295

[13] Baber U Dangas G Angiolillo DJ Cohen DJ Sharma SK Nicolas J Ticagrelor alone vs. ticagrelor plus aspirin following percutaneous coronary intervention in patients with non-ST-segment elevation acute coronary syndromes: TWILIGHT-ACS. *Eur Heart J.* (2020) 41:3533–45. 10.1093/eurheartj/ehaa670 33085967

[14] Dangas G Baber U Sharma S Giustino G Mehta S Cohen DJ Ticagrelor with or without aspirin after complex PCI. *J Am Coll Cardiol.* (2020) 75:2414–24. 10.1016/j.jacc.2020.03.011 32240761

[15] Escaned J Cao D Baber U Nicolas J Sartori S Zhang Z Ticagrelor monotherapy in patients at high bleeding risk undergoing percutaneous coronary intervention: twilight-HBR. *Eur Heart J.* (2021) 42:4624–34. 10.1093/eurheartj/ehab702 34662382

[16] Valgimigli M Mehran R Franzone A da Costa BR Baber U Piccolo R Ticagrelor monotherapy versus dual-antiplatelet therapy after PCI: an individual patient-level meta-analysis. *JACC Cardiovasc Interv.* (2021) 14:444–56. 10.1016/j.jcin.2020.11.046 33602441

[17] Franzone A McFadden EP Leonardi S Piccolo R Vranckx P Serruys PW Ticagrelor alone or conventional dual antiplatelet therapy in patients with stable or acute coronary syndromes. *Eurointervention.* (2020) 16:627–33. 10.4244/eij-d-20-00145 32482616

[18] Kim BK Hong SJ Cho YH Yun KH Kim YH Suh Y Effect of ticagrelor monotherapy vs ticagrelor with aspirin on major bleeding and cardiovascular events in patients with acute coronary syndrome: the TICO randomized clinical trial. *JAMA.* (2020) 323:2407–16. 10.1001/jama.2020.7580 32543684 PMC7298605

[19] Verheugt FWA Huber K Clemmensen P Collet J-P Cuisset T Andreotti F. Platelet P2y12 inhibitor monotherapy after percutaneous coronary intervention. *J Thromb Haemost.* (in press) (2022). 10.1055/s-0042-1755330 36584699

[20] Kogame N Guimarães PO Modolo R De Martino F Tinoco J Ribeiro EE Aspirin-free prasugrel monotherapy following coronary artery stenting in patients with stable cad: the ASET pilot study. *JACC Cardiovasc Interv.* (2020) 13:2251–62. 10.1016/j.jcin.2020.06.023 32950419

[21] Wiviott SD Braunwald E McCabe CH Montalescot G Ruzyllo W Gottlieb S Prasugrel versus clopidogrel in patients with acute coronary syndromes. *N Engl J Med.* (2007) 357:2001–15. 10.1056/NEJMoa0706482 17982182

[22] Wallentin L Becker RC Budaj A Cannon CP Emanuelsson H Held C Ticagrelor versus clopidogrel in patients with acute coronary syndromes. *N Engl J Med.* (2009) 361:1045–57. 10.1056/NEJMoa0904327 19717846

[23] Zettler ME Peterson ED McCoy LA Effron MB Anstrom KJ Henry TD Switching of adenosine diphosphate receptor inhibitor after hospital discharge among myocardial infarction patients: insights from the treatment with adenosine diphosphate receptor inhibitors: longitudinal assessment of treatment patterns and events after acute coronary syndrome (TRANSLATE-ACS) observational study. *Am Heart J.* (2017) 183:62–8. 10.1016/j.ahj.2016.10.006 27979043

[24] Antman EM Wiviott SD Murphy SA Voitk J Hasin Y Widimsky P Early and late benefits of prasugrel in patients with acute coronary syndromes undergoing percutaneous coronary intervention: a TRITON-TIMI 38 (trial to assess improvement in therapeutic outcomes by optimizing platelet inhibition with prasugrel-thrombolysis in myocardial infarction) analysis. *J Am Coll Cardiol.* (2008) 51:2028–33. 10.1016/j.jacc.2008.04.002 18498956

[25] Sibbing D Aradi D Jacobshagen C Gross L Trenk D Geisler T Guided de-escalation of antiplatelet treatment in patients with acute coronary syndrome undergoing percutaneous coronary intervention (TROPICAL-ACS): a randomised, open-label, multicentre trial. *Lancet.* (2017) 390:1747–57. 10.1016/s0140-673632155-4 28855078

[26] Cuisset T Deharo P Quilici J Johnson TW Deffarges S Bassez C Benefit of switching dual antiplatelet therapy after acute coronary syndrome: the topic (timing of platelet inhibition after acute coronary syndrome) randomized study. *Eur Heart J.* (2017) 38:3070–8. 10.1093/eurheartj/ehx175 28510646

[27] Kim CJ Park MW Kim MC Choo EH Hwang BH Lee KY Unguided de-escalation from ticagrelor to clopidogrel in stabilised patients with acute myocardial infarction undergoing percutaneous coronary intervention (TALOS-AMI): an investigator-initiated, open-label, multicentre, non-inferiority, randomised trial. *Lancet.* (2021) 398:1305–16. 10.1016/s0140-673601445-8 34627490

[28] Claassens DMF Vos GJA Bergmeijer TO Hermanides RS van ’t Hof AWJ van der Harst P A genotype-guided strategy for oral P2y inhibitors in primary PCI. *N Engl J Med.* (2019) 381:1621–31. 10.1056/NEJMoa1907096 31479209

[29] Moon JY Franchi F Rollini F Rivas Rios JR Kureti M Cavallari LH Role of genetic testing in patients undergoing percutaneous coronary intervention. *Expert Rev Clin Pharmacol.* (2018) 11:151–64. 10.1080/17512433.2017.1353909 28689434 PMC5771818

[30] Galli M Benenati S Capodanno D Franchi F Rollini F D’Amario D Guided versus standard antiplatelet therapy in patients undergoing percutaneous coronary intervention: a systematic review and meta-analysis. *Lancet.* (2021) 397:1470–83. 10.1016/s0140-673600533-x 33865495

[31] Tavenier AH Mehran R Chiarito M Cao D Pivato CA Nicolas J Guided and unguided de-escalation from potent P2y12 inhibitors among patients with acute coronary syndrome: a meta-analysis. *Eur Heart J Cardiovasc Pharmacother.* (2022) 8:492–502. 10.1093/ehjcvp/pvab068 34459481

[32] Mega JL Close SL Wiviott SD Shen L Hockett RD Brandt JT Cytochrome P-450 polymorphisms and response to clopidogrel. *N Engl J Med.* (2009) 360:354–62. 10.1056/NEJMoa0809171 19106084

[33] Gurbel PA Bliden KP Hiatt BL O’Connor CM. Clopidogrel for coronary stenting: response variability, drug resistance, and the effect of pretreatment platelet reactivity. *Circulation.* (2003) 107:2908–13. 10.1161/01.Cir.0000072771.11429.83 12796140

[34] Sibbing D Aradi D Alexopoulos D Ten Berg J Bhatt DL Bonello L Updated expert consensus statement on platelet function and genetic testing for guiding P2y receptor inhibitor treatment in percutaneous coronary intervention. *JACC Cardiovasc Interv.* (2019) 12:1521–37. 10.1016/j.jcin.2019.03.034 31202949

[35] Helten C Naguib D Dannenberg L Pöhl M Ayhan A Hohlfeld T Platelet function testing: dead or alive. *J Thromb Haemost.* (2018) 16:984–6. 29512292 10.1111/jth.13997

[36] Becker RC Bassand JP Budaj A Wojdyla DM James SK Cornel JH Bleeding complications with the P2y12 receptor antagonists clopidogrel and ticagrelor in the platelet inhibition and patient outcomes (PLATO) trial. *Eur Heart J.* (2011) 32:2933–44. 10.1093/eurheartj/ehr422 22090660

[37] Aradi D Gross L Trenk D Geisler T Merkely B Kiss RG Platelet reactivity and clinical outcomes in acute coronary syndrome patients treated with prasugrel and clopidogrel: a pre-specified exploratory analysis from the TROPICAL-ACS trial. *Eur Heart J.* (2019) 40:1942–51. 10.1093/eurheartj/ehz202 31226213

[38] Gurbel PA Tantry US Shuldiner AR Kereiakes DJ. Genotyping: one piece of the puzzle to personalize antiplatelet therapy. *J Am Coll Cardiol.* (2010) 56:112–6. 10.1016/j.jacc.2010.04.008 20471192

[39] Freynhofer MK Hein-Rothweiler R Haller PM Aradi D Dézsi DA Gross L Diurnal variability of on-treatment platelet reactivity in clopidogrel versus prasugrel treated acute coronary syndrome patients: a pre-specified TROPICAL-ACS sub-study. *Thromb Haemost.* (2019) 119:660–7. 10.1055/s-0038-1677549 30695790

[40] Lawton JS Tamis-Holland JE Bangalore S Bates ER Beckie TM Bischoff JM 2021 ACC/AHA/SCAI guideline for coronary artery revascularization: executive summary: a report of the American College of Cardiology/American Heart Association joint committee on clinical practice guidelines. *J Am Coll Cardiol.* (2022) 79:197–215. 10.1016/j.jacc.2021.09.005 34895951

[41] Huber K. Platelet antiaggregation after an acute coronary syndrome: what about the elderly? *Eur Heart J.* (in press) (2022). 10.1093/eurheartj/ehac405 36367685

[42] Koo BK Kang J Park KW Rhee TM Yang HM Won KB Aspirin versus clopidogrel for chronic maintenance monotherapy after percutaneous coronary intervention (HOST-EXAM): an investigator-initiated, prospective, randomised, open-label, multicentre trial. *Lancet.* (2021) 397:2487–96. 10.1016/s0140-673601063-1 34010616

